# Conservative management of triceps brachii muscle transection injury in an elite NCAA division I intercollegiate athlete: a case report and clinical review

**DOI:** 10.1016/j.xrrt.2024.12.008

**Published:** 2025-01-22

**Authors:** Guillermo Araujo-Espinoza, Al-Hassan Dajani, Varand Ghazikhanian, Alanna Salituro, Thomas J. Kremen

**Affiliations:** a Department of Orthopaedic Surgery, David Geffen School of Medicine at UCLA, Los Angeles, CA, USA; b Department of Diagnostic Radiology, David Geffen School of Medicine at UCLA, Los Angeles, CA, USA

**Keywords:** Triceps brachii, Muscle injury, Musculotendinous junction, Midsubstance muscle injuries, Nonoperative management, Overhead athlete, Return to plan

Injuries of the triceps include ruptures and avulsions of the triceps tendon-osseous insertion, tendon, musculotendinous junction, and muscle belly.^14^ Tendon and muscle-related injuries of the triceps brachii are uncommon,^1^ but can occur in association with sports participation.^2^^,^^7^^,^^8^^,^^9^^,^^11^^,^^12^^,^^14^^,^^16^

Rupture of the triceps tendon is rare, with only 262 cases reported in the literature.^4^ Rupture of the triceps muscle belly is even more rare, an extensive literature review of intramuscular injury to the triceps brachii revealed only 9 reported cases.^2^^,^^7^^,^^8^^,^^9^^,^^11^^,^^12^^,^^14^^,^^16^ The majority of cases result from direct trauma during active muscle contraction while participating in sports activities^2^^,^^6^^,^^7^^,^^12^^,^^10^^,^^15^ with only 2 cases involving indirect trauma.^2^^,^^9^ This study aims to describe the results of a significant dominant arm mid-substance injury to the triceps brachii muscle treated nonoperatively in an elite collegiate athlete.

## Case description

A 22-year-old right hand–dominant male competitive intercollegiate water polo player with no prior history of right brachium muscle injury was participating in riding a foil board while being towed behind a boat. During a maneuver, the tow line of the boat became tangled and circumferentially wrapped around his right brachium at which time he was dragged off the foil board and towed for several seconds until the boat stopped. He had immediate pain and was taken to the emergency room for evaluation of significant brachium swelling and ecchymosis; however, he was neurovascularly intact with no signs of acute compartment syndrome.

**Figure 1:**
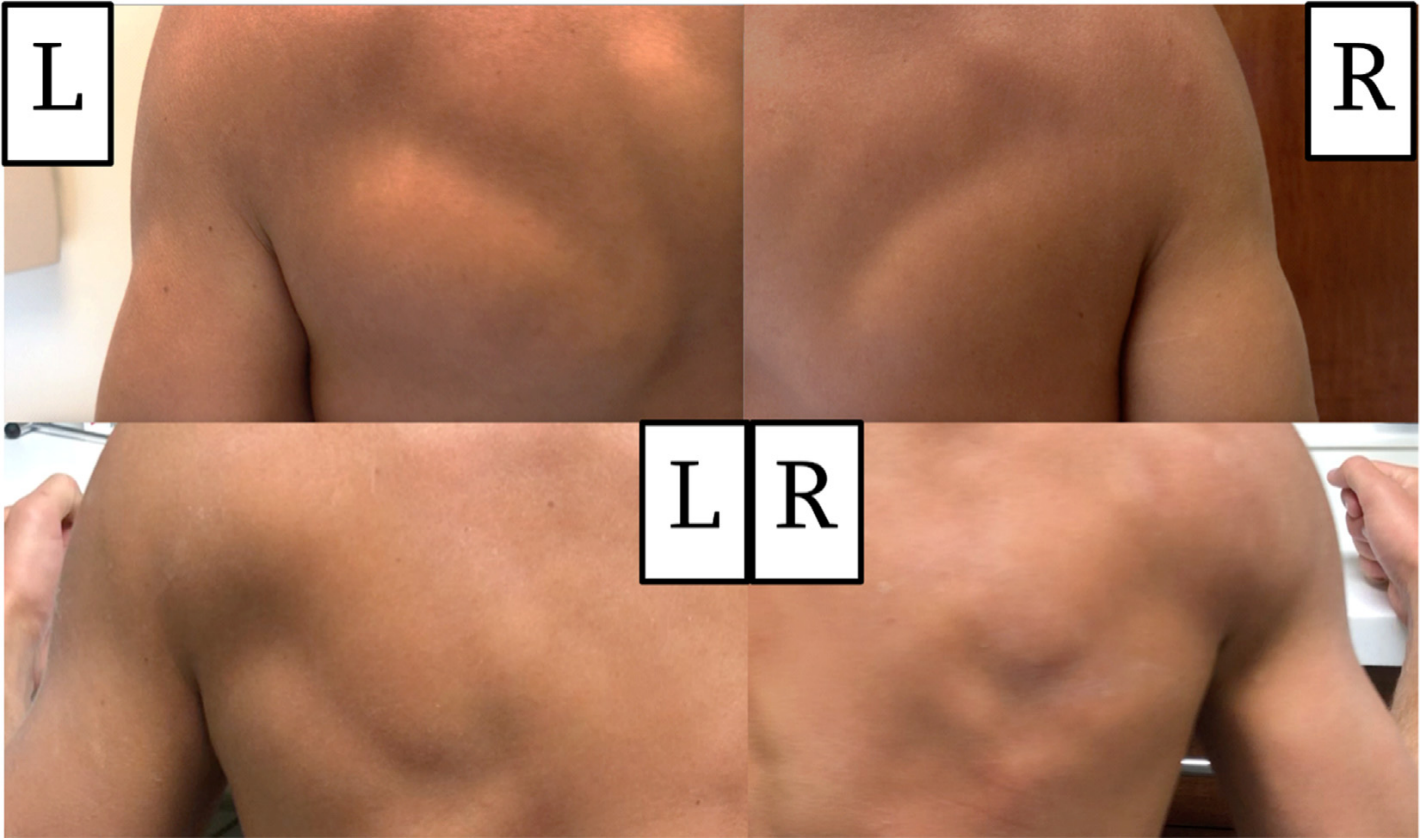
Posterior-lateral aspect of the left (L-uninjured) and right (R-injured) brachium 3 weeks (top) and (L-R) 16 weeks after injury (bottom). Of note, the 3-week image is without muscle activation and the 16-week image was captured during isometric contraction of the bilateral triceps muscles.

On follow-up in the senior author’s outpatient clinic 19 days after injury, his skin was noted to be intact. He did have erythema where the tow line was previously wrapped up, but, no ecchymosis was present. An asymmetry in the morphology of his anteromedial and posterior brachium contour was noted (Fig. 1). The patient was neurovascularly intact at the injured upper extremity with preserved active elbow flexion and extension, retaining ability to lift his arm overhead, albeit with some weakness due to pain. With his shoulder maximally forward flexed overhead, he could actively extend his elbow against gravity without issue and was able to generate 4 out of 5 strength such that he was able to push the examiner away with significant strength and perform 3 consecutive pushups with minimal effort. He had 5 out of 5 deltoid and rotator cuff muscle strength bilaterally including Jobe’s supraspinatus testing, infraspinatus testing (external rotation with the brachium adducted) and belly press testing. He had a palpable 2+ radial pulse and intact symmetric sensory function at the radial, ulnar, median, and axillary nerve distributions bilaterally. The patient had normal peripheral nerve motor function of the injured right upper extremity including intact finger flexion and extension, thumb flexion and extension, interossei strength, and anterior interosseous nerve motor function. Of note, the patient had undergone a previous right elbow ulnar collateral ligament repair of a distal soft tissue ligamentous avulsion 10 months before this boat tow line injury. After this ulnar collateral ligament repair, he had a loss of approximately 25° of supination at the right forearm; however, he had returned to full unrestricted water polo activities before the boat tow line injury to his right brachium. His pre-existing elbow range of motion deficit was unchanged compared to his exam before this boat tow line accident.

A few days before presenting to the senior author’s clinic, the patient had been evaluated by an outside physician who recommended expeditious surgery with allograft reconstruction of his coracobrachialis and triceps brachii. The patient was scheduled for surgery and came to see the senior author for a second opinion.

**Figure 2:**
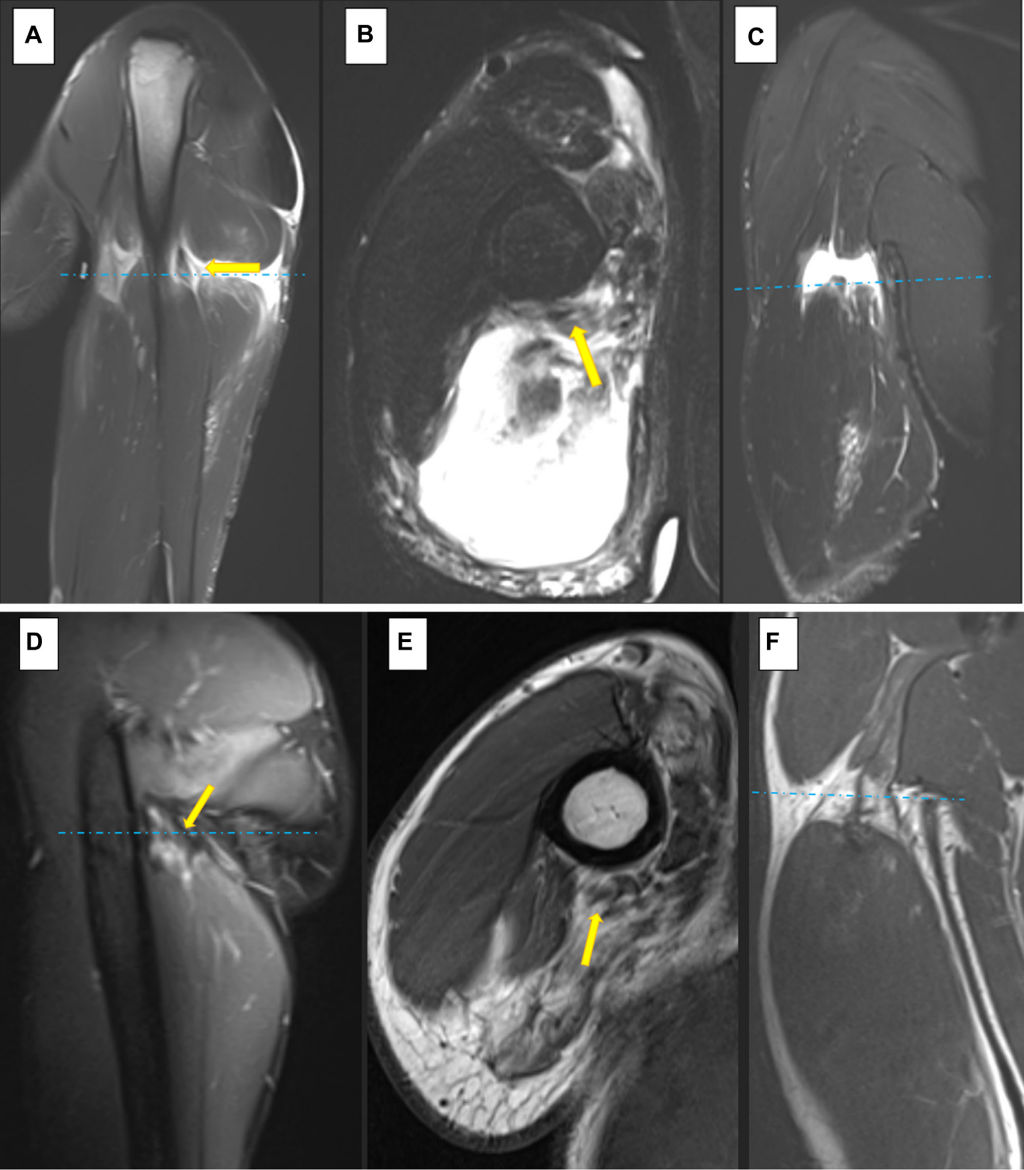
Right shoulder magnetic resonance (**A**) sagittal, (**B**) axial, and (**C**) coronal images 2 weeks after injury. Comparison right shoulder magnetic resonance (**D**) sagittal, (**E**) axial, and (**F**) coronal images 2.7 years after injury. () identify the radial nerve. () reflect the position of the axial slice on the corresponding sagittal and coronal images.

An magnetic resonance imaging (MRI) of the right brachium without contrast taken 2 weeks after injury reported a partial tear of the myotendinous junction of the lateral head and a complete tear of the proximal myotendinous junction of the long head of the triceps with a 2.3-cm gap. In addition, a high-grade partial tear of the proximal myotendinous junction of the long head with the conjoined short head of the biceps and coracobrachialis measuring 2.2 cm. No radial nerve damage was described (Fig. 2, *A-C*).

After review of the patient’s history, exam, and imaging studies, nonoperative management was recommended and the patient elected to proceed with conservative treatment. After physician approval, the athlete initiated a gradual rehabilitation program consisting of active range of motion exercises including concentric elbow flexion and extension. Initially this was against gravity, followed by low resistance 4-8 weeks after presentation. At 8 weeks after presentation, the patient advanced to submaximal triceps isometric exercises, increased eccentric triceps activity, and thrower-specific exercises. A throwing program was incorporated at 12-16, and water polo-specific exercises were permitted. At 5 months after injury, the patient had achieved all the prescribed functional milestones, including water polo-specific exercises. He had no appreciable deficits on exam and was cleared to return to participation in water polo activities. He returned to NCAA Division I intercollegiate competitive water polo at 7 months after injury. The patient’s performance during the 2 intercollegiate seasons before this boat tow line injury included a shooting percentage greater than 35%, an average of 3 or more assists per season, and 10 or more steals per season. After recovery from this boat tow line accident, during the patient’s subsequent 2 intercollegiate competition seasons he performed at the same level as he did before injury including a shooting percentage greater than 35%, averaging 3 or more assists and 10 or more steals per season.

Thirty months after injury, the patient underwent isometric strength testing using a MicroFET 2 hand-held dynamometer (Hoggan Scientific, Salt Lake, UT, USA) of the bilateral upper extremity. The test revealed slight strength differences between the injured (right) and noninjured (left) upper extremities, with the right biceps generating a mean force of 60.8 lbs compared to a mean of 63.0 lbs of force at the left biceps and the right triceps generating a mean force of 48.0 lbs compared to a mean of 50.1 lbs of force at the left triceps. Although this may not meet the needs of all throwing athletes,^10^^,^^17^ these results are insufficient to qualify as limb asymmetry^5^ (Table I). Despite these findings, as mentioned above, the patient successfully returned to their previous level of competition.

**Table I:** Upper-extremity handheld dynamometry testing results performed 30 months after injury.

	L		R		LSI	*P* value
	Mean	SD	Mean	SD		
Shoulder flexion	11.4 (25.0)	1.07	10.8 (23.9)	0.46	0.95	.01
Shoulder scaption	11.2 (24.6)	0.87	10.7 (23.7)	1.41	0.96	.13
Shoulder abduction	9.7 (21.3)	1.51	9.2 (20.3)	2.32	0.95	.18
Shoulder ER at 0°	11.0 (24.3)	1.07	11.2 (24.7)	1.32	1.02	.58
Bicep	28.6 (63.0)	2.76	27.6 (60.8)	1.70	0.97	.11
Tricep	22.7 (50.1)	3.52	21.8 (48.0)	3.28	0.96	.26
Grip at 0°	64.0 (141.1)	2.36	62.1 (136.9)	4.42	0.97	.04
Grip OH	59.0 (130.1)	8.46	58.4 (128.7)	8.13	0.99	.67

*ER*, external rotation; *LSI*, Limb Symmetry Index (R-injured value/L-uninjured value); *OH*, overhead; *SD*, standard deviation.

Grip overhead: Shoulder fully flexed with elbow fully extended; Grip at 0°: Elbow flexed at 90° with shoulder in neutral position (adducted, no rotation); lbs: force reported in kilograms, but measured in pounds (lbs).

Two-point-seven years after injury, the patient had a mild exacerbation of right shoulder pain in the setting of chronic superior labral tear. Follow-up MRI of the affected shoulder and injured brachium demonstrated chronic superior labral tear at the shoulder that was unchanged in appearance as well as changes consistent with healed muscle injury at the right brachium with extensive atrophy and fatty replacement of the triceps musculature with mild signs of proximal muscle denervation. Similar healing changes in the biceps as well as coracobrachialis were reported. The radial nerve was found to be intact (Fig. 2, *D*-*F*).

## Discussion

This report describes a case of a circumferential piano wire-type injury in a high-level intercollegiate water polo athlete with a significant muscle/muscle tendon junction injury that was successfully managed nonoperatively. There are a few reports on intramuscular rupture of the triceps: we only found 8 studies (9 patients)^2^^,^^7^^,^^8^^,^^9^^,^^11^^,^^12^^,^^14^^,^^16^ (Table II). Intramuscular rupture of the triceps brachii muscle is a rare condition usually observed in young, physically active individuals who engage in sports. These injuries appear to occur at higher rate in males than females given that 7 out of 9 prior cases involved male patients. The reported cases included adults between the ages of 20-48, with one exception being a 13-year-old boy waterskiing.^14^

**Table II:** Summary table of previous triceps brachii muscle rupture reports divided by type for treatment (nonoperative or surgery).

Treatment	Study	Age	Sex	Injury mechanism	Diagnostic imaging	Triceps head rupture involvement			Follow-up	Time to RTS/sport	Result
						Long head	Lateral	Medial			
Nonoperative	Post^12^	48	M	Abrupt arm extension against fixed surface	None	NR. Clinical: “Partial posteromedial tear”			2 y	NR	Slight loss of extension power
	Aso (Case 2)^2^	36	F	Serving overhead while playing volleyball	X-Ray, CT	NR. CT: “Remarkable posteromedial increase volume”.			1.6 y	NR	Complete function
	O’Driscoll^9^	25	M	Paddling with maximum effort while white-water kayaking	None	Clinical: Complete	None	None	10 y	3 y (slight endurance weakness)/Kayaking	Equivalent Isometric strength bilaterally with associated 5%-10% decrease in endurance strength
	Sheps^14^	13	M	Initially dragged while waterskiing, then reinjured while golfing 9 mo later	US, MRI	Partial	None	None	∼2 y	NR	No functional deficits per patient, however Cybex strength testing demonstrates significant extension and pronation deficits
	Present	21	M	Direct trauma while foil boarding as rope wrapped patient's arm and dragged patient briefly	MRI	Complete	Partial	Complete	2.5 y	7 mo/Water polo	Complete function
Surgery	Singh^16^	31	M	Struck on back of arm by opponent while playing ice hockey, then collided with barrier while holding stick at waist height	MRI	Complete	Complete	Complete	6 mo	6 mo/Ice Hockey	Complete function
	Kaseta^7^	42	M	Direct trauma in the posterior aspect of the arm	X-Ray	Complete	Partial	Complete	5.3 y	NR	Complete function
	Penhallow^11^	35	M	Struck by a box while unloading cargo at port	None	Complete	Partial	Partial	1 mo	NR	Complete function
	Montgomery^8^	20	M	Head-on impact to posterior arm by tailboard of truck	None	Complete	Complete	Complete	2 mo	NR	Complete function
	Aso (Case 1)^2^	28	M	Suffered blow to arm by opponent in Kendo	X-Ray	None	None	Partial (Already scarred)	1.5 y	NR	Complete function

*NR*, not reported; *MRI*, magnetic resonance imaging; *RTS*, return to sport; *US*, ultrasound; *CT*, computed tomography.

The majority of past studies describe direct trauma during physical activity and muscle contraction as the primary mechanism of triceps brachii muscle injury. These injuries resulted from participation in ice hockey,^16^ martial arts,^2^ water sports,^14^ heavy labor,^8^ daily activities,^7^ or accidents.^11^ One indirect case of rupture was reported due to the patient performing forward strokes with maximal effort while kayaking; the author inferred a fatigue mechanism.^9^ Indirect trauma has also been observed in a middle-aged woman whose arm was extended against resistance.^2^^,^^12^ The mechanism of injury in our case is similar to a previous case of a 13-year-old boy dragged by a tow line while waterskiing.^14^

Patients with intramuscular rupture of the triceps typically present with a distinct medical history, and examination often reveals pain described as mild^7^ or severe,^8^ diffuse swelling, presence of ecchymosis^2^^,^^7^^,^^8^^,^^16^ or lack of ecchymosis ^11^ as in our case. Possible findings also include weakness during^7^^,^^14^^,^^16^ or an inability to fully extend the elbow,^8^^,^^12^ primarily with almost complete or total rupture. A noticeable sulcus or altered muscle contour in the posterior part of the arm may also confirm the diagnosis.^9^^,^^11^^,^^12^ It is possible to find a palpable mass that is more prominent during contraction and extension of the arm which disappears during relaxation.^2^^,^^11^

MRI has been used to identify intramuscular rupture.^14^^,^^16^ However, results should be interpreted cautiously, as illustrated in Singh’s study when a negative MRI was initially reported, and a rupture of all 3 triceps heads was discovered during surgical exploration.^16^ In Kaseta’s report, the MRI imaging was deemed unnecessary due to the extent of damage seen on clinical examination, and direct surgical intervention was proposed instead,^7^ similar to older nonoperative reports.^9^^,^^12^ However, in other cases, X-rays were used to rule out triceps avulsion.^2^ Moreover, ultrasound^14^ or computed tomography scans^2^ were used to confirm the diagnosis and determinelesion size.

Injury ranges from partial to complete rupture, showing a good prognosis for patients overall. Out of the 10 cases reported in the literature (including the present report), 2 cases involved a complete rupture of all 3 heads of the triceps,^8^^,^^16^ while some cases had an almost complete rupture with 50% of the lateral head remaining^7^^,(present)^. Two cases had only the long head affected^9^^,^^14^ and 2 other cases had a lesion only of the medial head.^2^^,^^11^ Additionally, 2 cases did not report the extension of the lesion.^2^^,^^12^

Literature review demonstrates that the treatment of triceps brachii injuries is variable. Of the 10 known cases to date, half were managed conservatively, and the other half underwent surgery, indicating a lack of consensus on the best treatment option for intramuscular rupture of the triceps brachii muscle. One case report of a patient managed conservatively demonstrates equal isometric strength after 9 years plus a 5%-10% endurance decrease on the affected side.^9^ Another report of conservative management shows a deficit in extension, pronation, and peak torque on extension in Cybex testing without functional weakness or significant disability.^14^ The oldest reports on triceps muscle rupture suggest mandatory surgery in complete tears. However, these older reports on triceps muscle rupture lack objective measurements or functional outcome measures to support surgical management.^8^^,^^11^ Although there is no definitive clinical evidence to guide the decision for conservative versus surgical treatment of intramuscular rupture of the triceps brachii muscle, physicians should consider the extent of the injury.^16^ A threshold of 50% involvement of triceps brachii muscle tissue has been suggested as an indication for surgical intervention.^7^ However, others have noted that injury at the musculotendinous junction or the muscle belly, may best be treated conservatively.^6^^,^^9^^,^^15^^,^^13^ Furthermore, the functional demands of the patient deserve consideration. O'Driscoll has recommended nonoperative treatment for patients who do not need significant endurance strength in elbow extension, although the potential benefit of surgical repair remains uncertain.^9^

While surgical reconstruction with an allograft was considered in this patient and even recommended by a consulting physician, this injury was treated conservatively and resulted in excellent patient function. Other authors have also noted that nonsurgical treatment of intramuscular triceps ruptures is a viable treatment option, despite reports of a slight decrease in elbow extension power^12^^,^^14^ and endurance.^9^ Despite these reported minor functional deficits, the overall results have been reported as good^9^^,^^12^^,^^14^ or excellent^2^ following conservative treatment including this current case report. Also noteworthy, in 1 reported case treated surgically 4 weeks after injury, the separated medial origin of the triceps was found to be invested with significant scar tissue, and, as a result surgical repair was deemed unnecessary intraoperatively.^2^

In the present case report, the patient’s ability to perform activities of daily living was restored within 3 weeks of injury, similar to Sheps et al, at 1 week after nonoperative treatment.^14^ Surgical intervention has been associated with the restoration of activities of daily living at 4,^11^ 8,^8^ and 16 weeks.^7^ Although there is a scarcity of reports among competitive athletes, return to sport has been reported at 6 months after surgery,^16^ which is consistent with our current case returning to competitive waterpolo at 7 months after nonoperative care.

Conservative treatment was preferred in this case because the patient had recovered excellent function within 3 weeks following the injury, and surgery in this area could potentially damage several neurovascular structures. Furthermore, outcomes after surgical management of mid-substance muscle belly injuries remain unpredictable and are the subject of ongoing debate, as previously discussed.

This case illustrates the importance of physical exam over imaging and highlights the concept that musculoskeletal medicine specialists should treat patients rather than significant imaging findings, even when imaging findings are highly concerning. This report also demonstrates the ability of patients to recover from significant muscle injury with excellent function when managed nonoperatively, even in an elite intercollegiate athlete. As has been the experience among musculoskeletal oncologic surgeons who routinely perform en bloc resections including muscle tissues, there are soft tissue attachments between adjacent muscles that allow for side-to-side interfascial “healing” and interposition of fibrotic reparative tissue despite the significant structural injury.^3^ It is likely that this interfascial healing combined with the formation of fibrotic reparative tissue spanning the muscle defect associated with this injury contributed significantly to the excellent retained function of the injured muscle in our patient. Muscle fibrosis can impair muscle function while also serving as a link between the 2 ends of a torn muscle belly. Furthermore, strength is related to cross-sectional area over the length of a muscle. In this case, a loss of cross-sectional area due to fibrosis was over a limited length of the muscle belly. The remainder of the muscle belly length remained intact.

## Conclusion

Intramuscular rupture/muscle–tendon junction injuries of the triceps brachii muscle are uncommon injuries that can present with varied clinical findings. These injuries are typically observed in young and healthy individuals participating in sports activities. Only 10 cases have been documented in the literature, including this one. Currently, there is no consensus on whether conservative or surgical treatment yields better outcomes. As presented in this case report, nonoperative management of a dominant arm significant mid-substance triceps brachii muscle injury can allow for full restoration of function and no significant impairment in performance, even in an elite throwing athlete.

## Disclaimers:

Funding: Dr. Thomas J. Kremen Jr. is supported by the 10.13039/100000738US Dept of Veterans Affairs grant number IK2BX005199.

Conflicts of interest: Research support for Thomas J. Kremen Jr. is provided by US Department of Veterans Affairs grant number IK2BX005199. The other authors, their immediate families, and any research foundations with which they are affiliated have not received any financial payments or other benefits from any commercial entity related to the subject of this article.

Patient consent: Obtained.

## References

[1] Anzel S.H., Covey K.W., Weiner A.D., Lipscomb P.R. (1959). Disruption of muscles and tendons; an analysis of 1, 014 cases. Surgery.

[2] Aso K., Torisu T. (1984). Muscle belly tear of the triceps. Am J Sports Med.

[3] Burke Z.D.C., Blumstein G.W., Zoller S.D., Park H.Y., Bernthal N.M. (2018). Reconstructive science in orthopedic oncology. Tech Orthop.

[4] Dunn J.C., Kusnezov N., Fares A., Rubin S., Orr J., Friedman D. (2017). Triceps tendon ruptures: a systematic review. Hand.

[5] Felix I., Dines D., Dines J. (2021). Interval return to play programs for the tennis athlete. Curr Rev Musculoskelet Med.

[6] Harris P.C., Atkinson D., Moorehead J.D. (2004). Bilateral partial rupture of triceps tendon: case report and quantitative assessment of recovery. Am J Sports Med.

[7] Kaseta M.K., Queen R.M., Moorman C.T. (2010). Traumatic closed transection of the triceps brachii: a case report. J Surg Orthop Adv.

[8] Montgomery A.H. (1920). Two cases of muscle injury. Surg Clin Chicago.

[9] O'Driscoll S.W. (1992). Intramuscular triceps rupture. Can J Surg.

[10] Otley T., Myers H., Lau B.C., Taylor D.C. (2022). Return to sport after shoulder stabilization procedures: a criteria-based testing continuum to guide rehabilitation and inform return-to-play decision making. Arthrosc Sports Med Rehabil.

[11] Penhallow D.P. (1919). Report of a case of ruptured triceps due to direct violence. NY Med J.

[12] Post M., Febiger L. (1988). The shoulder surgical and nonsurgical management.

[13] van Riet R.P., Morrey B.F., Ho E., O'Driscoll S.W. (2003). Surgical treatment of distal triceps ruptures. J Bone Joint Surg Am.

[14] Sheps D., Black G.B., Reed M., Davidson J.M. (1997). Rupture of the long head of the triceps muscle in a child: case report and review of the literature. J Trauma.

[15] Sierra R.J., Weiss N.G., Shrader M.W., Steinmann S.P. (2006). Acute triceps ruptures: case report and retrospective chart review. J Shoulder Elbow Surg.

[16] Singh R.K., Pooley J. (2002). Complete rupture of the triceps brachii muscle. Br J Sports Med.

[17] Wilk K.E., Bagwell M.S., Davies G.J., Arrigo C.A. (2020). Return to sport participation criteria following shoulder injury: a clinical commentary. Int J Sports Phys Ther.

